# Molecular Characterization and Viral Origin of the First Dengue Outbreak in Xishuangbanna, Yunnan Province, China, 2013

**DOI:** 10.4269/ajtmh.14-0044

**Published:** 2015-08-05

**Authors:** Xiaofang Guo, Henglin Yang, Chao Wu, Jinyong Jiang, Jianhua Fan, Hongbin Li, Jin Zhu, Zhonghua Yang, Yuanyuan Li, Hongning Zhou, Jiusong Zhang

**Affiliations:** Yunnan Provincial Key Laboratory of Arbo Infectious Disease Control Research (Preparing), Yunnan Institute of Parasitic Diseases, Yunnan, People's Republic of China; State Key Laboratory of Pathogen and Biosecurity, Beijing Institute of Microbiology and Epidemiology, Beijing, People's Republic of China; Division of Parasitic Diseases Prevention and Control, Xishuangbanna Center for Disease Control and Prevention, Yunnan, People's Republic of China

## Abstract

In August 2013, Xishuangbanna, Yunnan Province, China, had its first dengue outbreak. Dengue virus (DENV) RNA detection in sera or viral isolates revealed that all 222 autochthonous patients detected and three Chinese travelers from Laos (imported cases) were positive for DENV-3 serotype, while DENV-1 and DENV-4 were detected in travelers from Myanmar and Thailand during the outbreak. For 33 suspected dengue cases collected before the outbreak, two imported cases from Laos and nine residents living in Laos (Laotian cases) were positive for DENV-3. Further, a random subset of 33 positive cases for DENV-3 was sequenced for the full envelope gene of DENV. Phylogenetic analysis showed that all of the 25 autochthonous cases sequenced were grouped into the same clade, genotype II of DENV-3, with imported cases from Laos and Laotian cases. These results suggest that the genotype II of DENV-3 was associated with the outbreak and may have originated from the virus circulating in Laos.

Dengue is a mosquito-borne disease caused by the dengue virus (DENV). DENV is a member of the genus *Flavivirus* of the family Flaviviridae and has four distinct serotypes (DENV-1, DENV-2, DENV-3, and DENV-4).^1^ The disease is prevalent throughout the tropical and subtropical regions of the world and is the most rapidly spreading one, with a 30-fold increase in global incidence over the past 50 years. It is estimated that 390 million dengue infections occur worldwide annually.^2^ More than half of the world's population lives in dengue-endemic regions; the disease is especially prevalent in southeast Asia, the west Pacific Ocean regions, southern Africa, and Latin America. The disease spectrum ranges from asymptomatic, mild undifferentiated fever and classic dengue fever to severe dengue hemorrhagic fever and dengue shock syndrome.

The first outbreak of dengue in mainland China occurred in Foshan of Guangdong Province in 1978. Since then, disease outbreaks have been recorded sequentially in the Hainan, Guangxi, Fujian, and Zhejiang provinces.^3^ The Yunnan Province, locating in the southwest of China, has a tropical to subtropical climate. Since the establishment of a dengue surveillance system in 2005, imported dengue cases have been reported annually in the area, but no dengue outbreaks had been reported before 2013.^4^,^5^ In 2013, a dengue outbreak was reported in Xishuangbanna, which is located in the southwestern Yunnan Province and borders with Laos and Myanmar, where dengue is endemic. Here, we report the results of the detection and serotyping of the DENV that caused the outbreak. Phylogenetic analyses based on envelope (*E*) gene sequences of the virus were also performed to understand the genetic characterization and potential source of the virus.

The study was performed after consultation with the patients and receipt of written consent. All study-related information was acquired and used anonymously. The Institutional Review Board of the Yunnan Institute of Parasitic Diseases (YIPD) approved the research involving human subjects.

The dengue cases were mainly diagnosed based on dengue fever symptoms, including fever, headache, retro-ocular and joint pain, rash, lymphadenopathy, and positive DENV nonstructural protein (NS) 1 antigen detected with the Dengue Ag Rapid Test (CTK Biotech, Inc., San Diego, CA), which is the standard method used to rapidly detect antigens in DENV infections. From August 15 to November 25, 2013, the Xishuangbanna Center for Disease Control (CDC) confirmed a total of 1,254 autochthonous dengue cases, who had never traveled abroad or to other areas of China. Most infections (80% of cases) occurred between the ages of 15 and 60 years, and no deaths associated with the outbreak were recorded.

Of the 1,254 cases, 360 autochthonous cases were selected mainly based on the onset date of the disease. In addition, eight imported cases, three from Laos (two in August and one in September), three from Myanmar (August), and two from Thailand (August) were confirmed through the dengue surveillance system. Before the outbreak, 33 acute-phase plasma samples were obtained in June 2013 from suspected dengue cases with the dengue fever symptoms, including four Chinese travelers from Houayxay County and Vientiane, Laos (imported cases), and 29 residents living in the Houayxay and Luang Prabang counties in the north of Laos (Laotian cases), where the Joint Control Project of Malaria and Dengue in China's frontier areas adjacent to Laos was under implementation. A total of 401 cases were included in this study. The map of the sampling locations is shown in Figure 1

**Figure 1.**
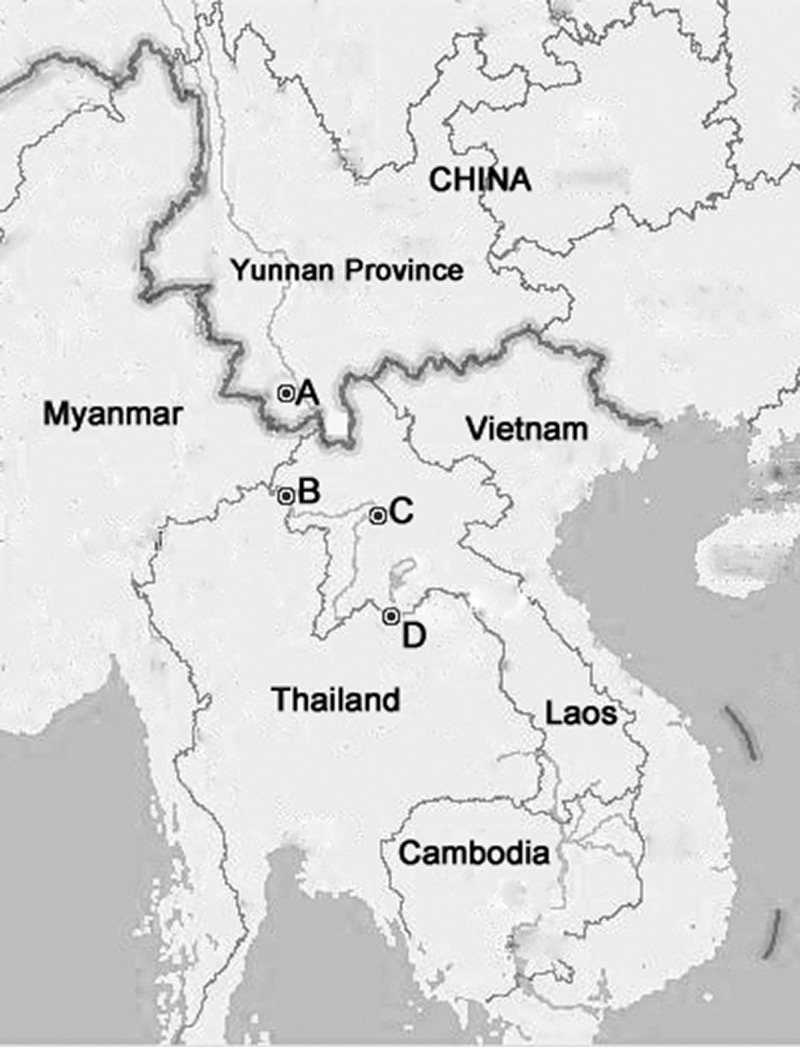
Map of Yunnan Province and neighboring countries. (**A**) (Jinghong City, Xishuangbanna, Yunnan Province, China) shows the location of dengue outbreak. (**B**) (Houayxay County, Bokeo Province, Laos) and (**D**) (Vientiane, Laos) show the locations of imported dengue cases. (**B**) and (**C**) (Luang Prabang County, Luang Prabang Province, Laos) show the locations of Laotian cases. Imported case refers to Chinese traveler, who felt ill in Laos, and then came back to China. Laotian case refers to Laotian, who was infected in Laos, and had never traveled abroad.

.

RNA was extracted from the plasma samples using Column Viral RNAout (TIANDZ, Beijing, China), and was reverse transcribed using random hexamers to synthesize the first-strand complementary DNA (cDNA). The DENV was detected and typed using a semi-nested reverse transcription polymerase chain reaction (RT-PCR) with universal and serotype-specific primers.^6^ The full *E* gene of DENV was amplified as described by Liang and others.^7^ The PCR products were directly sequenced using an automated DNA sequencer (ABI PRISM 3730, Carlsbad, CA). The nucleotide sequences of the viruses were analyzed to determine the sequence identity using the Lasergene software package (DNASTAR, Madison, WI).

Viral isolation was conducted on the imported cases selected. In brief, the serum samples were 10-fold diluted with RPMI-1640 medium containing 2% fetal calf serum and cultured with *Aedes albopictus* C6/36 cells for 7–10 days. Cultures were examined daily for evidence of a virally induced cytopathic effect (CPE). Cultures without CPE were blind passaged three times.

Multiple sequence alignments and phylogenetic analysis were carried out using the MEGA 5.0 program (http://www.megasoftware.net) based on the sequences generated in this study, and the reference sequences were downloaded from GenBank including the prototype and genotyping strains of DENV-3 and representative strains from China, southeast Asia, south Asia, Oceania, North America, South America, and Africa. Multiple sequence alignment was carried out in ClustalW of the MEGA 5.0 program. Phylogenetic trees were constructed using the maximum likelihood statistical method based on the Tamura-Nei model. The robustness of the resulting tree was established by bootstrap analysis with 1,000 replications.

The results of the DENV RT-PCR and viral isolation are summarized in Table 1. The RT-PCR results showed that 222 of the 360 autochthonous cases were positive for DENV-3, and the other 138 cases were negative for DENV. The three cases imported from Laos were positive for DENV-3, and each one of three imported cases from Myanmar was positive for DENV-1 and DENV-4, while another case from Myanmar and the two cases from Thailand were negative for DENV. For viral isolation, one DENV-4 strain was obtained from the two imported cases from Thailand. For the 33 suspected dengue cases collected before the outbreak, two DENV-3 strains and one DENV-4 strain from four imported cases from Laos were obtained, and nine of the 29 Laotian cases were positive for DENV-3 by RT-PCR.

A random subset of 33 positive cases for DENV-3 including 25 autochthonous cases selected from the different month of the outbreak, four cases imported from Laos during or before the outbreak, and four Laotian dengue patients from two counties in northern Laos were selected and sequenced for the 1,479-bp *E* gene of DENV (Table 1, Supplemental Table 1).

The phylogenetic tree based on the full *E* sequences of DENV-3 is shown in Figure 2A

**Figure 2.**
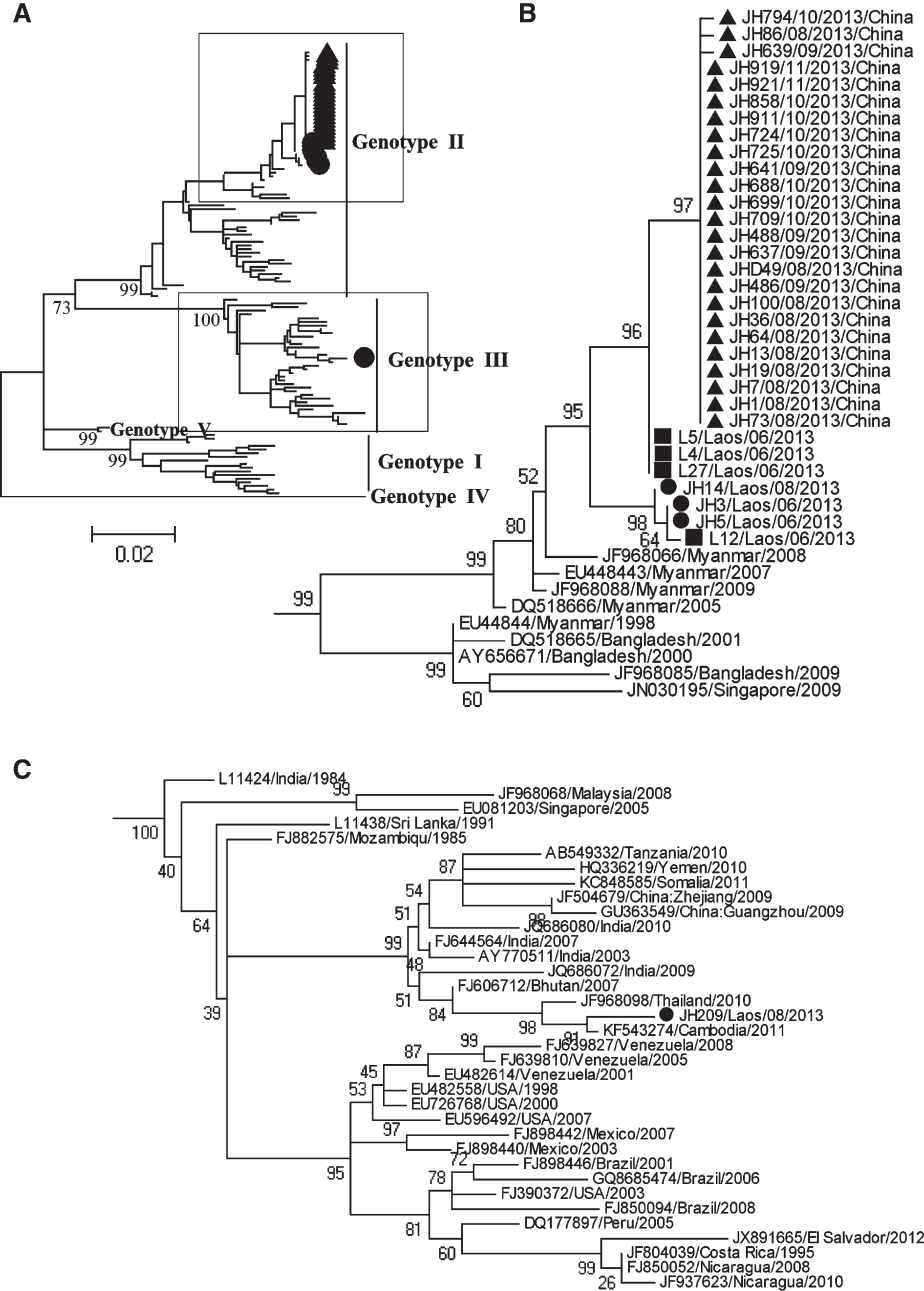
Phylogenetic analysis based on the full envelope protein sequences of dengue virus (DENV)-3 from autochthonous, imported, and Laotian patients. The phylogenetic tree was constructed using the maximum likelihood statistical method in the MEGA 5.0 program. Bootstrap values were obtained from 1,000 replicates. (**A**) Topological figure of integral phylogenetic tree based on 99 envelope gene sequences from different geographic regions available in GenBank database and 33 new sequences obtained in this study. (**B**) Clade of genotype II, which is magnified from “**A**,” including 32 new sequences. (**C**) Clade of genotype III, which is magnified from “**A**,” including another new sequence. Each DENV sequence is denoted by its GenBank accession number, country of origin, and year of isolation. Autochthonous, Laotian, and imported sequences obtained in this study are denoted by “▲”, “■”, and “●” respectively.

. A total of 123 DENV-3 sequences were included in this analysis. These sequences included 33 new sequences from this study with the GenBank accession numbers KF816147–KF816164 and KM651768–KM651782, and 90 sequences that represented different geographies, genotypes, and years of isolation (Supplemental Table 2). The tree showed five distinct clades corresponding to genotype I–V of DENV-3. The 25 autochthonous sequences, together with three imported and four Laotian sequences collected from the northern Laos were grouped into the same cluster, which belonged to the genotype II (Thailand genotype) of DENV-3 (Figure 2B), while one imported sequence from the middle of Laos on August 20, 2013, was clustered into the genotype III group known as the Indian subcontinent genotype of DENV-3 (Figure 2C). The 32 newly obtained genotype II sequences were most closely related to the sequences collected from Myanmar in 2005 and 2009. No genetic clustering was associated with the onset date of the disease. In the DENV-3 genotype II samples, the autochthonous cases had the highest nucleotide sequence identities (98.9–99.7%) with the newly obtained Laotian sequences. The genotype III group included sequences from America and Asia. The new genotype III sequence was most closely related to the 2011 Cambodia sequence; these two sequences shared 99.1% nucleotide sequence identity.

We detected DENV-3 RNA in samples from autochthonous cases and in samples that were collected during or before the outbreak from cases imported from Laos and Laotian. The phylogenetic analysis indicated that the dengue outbreak that occurred in Xishuangbanna in 2013 was primarily caused by genotype II of the DENV-3, which had the closest relation with the DENV from northern Laos. Our results suggested that the DENV that caused the 2013 autochthonous outbreaks most likely originated from the DENV circulating in the north of Laos. Importantly, we also detected the DENV-3 genotype III in a patient who had returned from Laos. This genotype has newly emerged in southeast Asian countries and has frequently caused dengue hemorrhagic fever in Sri Lanka since 1989.^8^,^9^

Moreover, DENV-1 and DENV-4 were detected from travelers to Myanmar, and DENV-4 was isolated from both a traveler to Laos in June 2013 and a traveler to Thailand in August 2013. In 2009, we first isolated a DENV-2 strain from a traveler to Laos.^10^ All of the evidence suggests that multiple DENV serotypes are endemic in the neighboring countries. The autochthonous dengue outbreak highlights the ease by which an imported DENV strain can spread and cause an outbreak. With increased trade and international travel in the Greater Mekong Subregion, it is inevitable that DENVs will be introduced into China from neighboring countries. A sound laboratory-based disease surveillance system and integrated vector management are required in the border regions of Yunnan Province so that future disease outbreaks and spread to other regions can be prevented.

## Supplementary Material

Supplemental Tables.

## Figures and Tables

**Table 1:** Results of DENV RNA detection, viral isolation, and sequencing in autochthonous, imported, and Laotian patients

	Case classification	Positive for RT-PCR* (serotype)	Positive for viral isolation* (serotype)	No. of DENV-3 *E* gene sequencing
During the outbreak	Autochthonous	222/360 (all DENV-3)	ND	25
	Imported from Laos	3/3 (all DENV-3)	ND	2
	Imported from Myanmar	2/3 (DENV-1, DENV-4 each one)	ND	ND
	Imported from Thailand	0/2	1/2 (DENV-4)	ND
Before the outbreak	Laotian	9/29 (all DENV-3)	ND	4
	Imported from Laos	ND	3/4 (two DENV-3, one DENV-4)	2

DENV = dengue virus; ND = not done.

* Number of positive/total number detected.
