# Supplementary material for: Molecular Characterization and Viral Origin of the First Dengue Outbreak in Xishuangbanna, Yunnan Province, China, 2013
Source: Am J Trop Med Hyg. 2015 Aug 5;93(2):390–3. doi: 10.4269/ajtmh.14-0044 (PMC4530767; doi:10.4269/ajtmh.14-0044)
Supplement: Supplementary file 1 [file SD8.pdf]

Supplemental Table 1

Basic information of Chinese and Laos samples selected for sequencing of the envelope protein gene, 2013

Patient classification, strain	Patient's nationality	Location of infection	Date of onset	Date of sampling	Isolation source
Local cases					
JH1/08/2013/China	Chinese	China:Jinghong city	August 10, 2013	August 15, 2013	Serum
JH7/08/2013/China	Chinese	China:Jinghong city	August 15, 2013	August 16, 2013	Serum
JH13/08/2013/China	Chinese	China:Jinghong city	August 14, 2013	August 17, 2013	Serum
JH19/08/2013/China	Chinese	China:Jinghong city	August 16, 2013	August 17, 2013	Serum
JH36/08/2013/China	Chinese	China:Jinghong city	August 15, 2013	August 17, 2013	Serum
JH64/08/2013/China	Chinese	China:Jinghong city	August 15, 2013	August 18, 2013	Serum
JH73/08/2013/China	Chinese	China:Jinghong city	August 17, 2013	August 18, 2013	Serum
JH86/08/2013/China	Chinese	China:Jinghong city	August 15, 2013	August 18, 2013	Serum
JH100/08/2013/China	Chinese	China:Jinghong city	August 18, 2013	August 19, 2013	Serum
JHD49/08/2013/China	Chinese	China:Jinghong city	August 19, 2013	August 22, 2013	Serum
JH486/09/2013/China	Chinese	China:Jinghong city	September 19, 2013	September 20, 2013	Serum
JH488/09/2013/China	Chinese	China:Jinghong city	September 18, 2013	September 20, 2013	Serum
JH637/09/2013/China	Chinese	China:Jinghong city	September 28, 2013	September 29, 2013	Serum
JH639/09/2013/China	Chinese	China:Jinghong city	September 28, 2013	September 29, 2013	Serum
JH641/09/2013/China	Chinese	China:Jinghong city	September 26, 2013	September 30, 2013	Serum
JH688/10/2013/China	Chinese	China:Jinghong city	October 1, 2013	October 2, 2013	Serum
JH699/10/2013/China	Chinese	China:Jinghong city	October 1, 2013	October 3, 2013	Serum
JH709/10/2013/China	Chinese	China:Jinghong city	October 3, 2013	October 4, 2013	Serum
JH724/10/2013/China	Chinese	China:Jinghong city	October 3, 2013	October 6, 2013	Serum
JH725/10/2013/China	Chinese	China:Jinghong city	October 2, 2013	October 6, 2013	Serum
JH794/10/2013/China	Chinese	China:Jinghong city	October 12, 2013	October 13, 2013	Serum
JH858/10/2013/China	Chinese	China:Jinghong city	October 18, 2013	October 20, 2013	Serum
JH911/10/2013/China	Chinese	China:Jinghong city	October 31, 2013	November 3, 2013	Serum
JH919/11/2013/China	Chinese	China:Jinghong city	November 3, 2013	November 5, 2013	Serum
JH921/11/2013/China	Chinese	China:Jinghong city	November 1, 2013	November 5, 2013	Serum
Imported cases					
JH14/Laos/08/2013	Chinese	Laos:Houayxay	August 13, 2013	August 17, 2013	Serum
JH209/Laos/08/2013	Chinese	Laos:Vientiane	August 13, 2013	August 20, 2013	Serum
JH3/Laos/06/2013	Chinese	Laos:Houayxay	June 23, 2013	June 26, 2013	Cell supernatant
JH5/Laos/06/2013	Chinese	Laos:Houayxay	June 22, 2013	June 26, 2013	Cell supernatant
Laotian cases					
L4/Laos/06/2013	Lao	Laos:Luang Prabang county	NA	June 2013	Serum
L5/Laos/06/2013	Lao	Laos:Luang Prabang county	NA	June 2013	Serum
L12/Laos/06/2013	Lao	Laos:Houayxay county	NA	June 2013	Serum
L27/Laos/06/2013	Lao	Laos:Houayxay county	NA	June 2013	Serum

NA = not applicable.

Supplemental Table 2

Information on DENV-3 sequences used in phylogenetic analysis

Continent	Geography	DENVs strains in this study	GenBank	Collection year	Strain
Asian	China	JF504679/China:Zhejiang/2009	JF504679	2009	ZJYW2009
		KC261634/China:Guangzhou/2012	KC261634	2012	GZ/10476/2012
		JN009098/China:Guangzhou/2010	JN009098	2010	10/GZ/10549
		GU363549/China:Guangzhou/2009	GU363549	2009	GZ1D3
		AF317645/China:Guangxi/1980	AF317645	1980	80-2
	Myanmar	JF968088/Myanmar/2009	JF968088	2009	D3/Myanmar/0911aTw
		JF968066/Myanmar/2008	JF968066	2008	D3/Myanmar/0810aTw
		EU448443/Myanmar/2007	EU448443	2007	0707aTw
		DQ518666/Myanmar/2005	DQ518666	2005	Myan0508a/Tw
		EU44844/Myanmar/1998	EU44844	1998	D3/Myanmar/9809aTw
		DQ518655/Myanmar/1996	DQ518655	1996	Viet9609a/Tw
	Thailand	JF968098/Thailand/2010	JF968098	2010	D3/Thailand/1008aTw
		JN575576/Thailand/2010	JN575576	2010	Thailand 2010
		JF968084/Thailand/2009	JF968084	2009	D3/Thailand/0910aTw
		JF812104/Thailand/2006	JF812104	2006	TH/DB054/2006
		JQ993230/Thailand/2004	JQ993230	2004	Thailand_KPP_KDS00998_NC06_ Schol_S_11_2004
		AY676383/Thailand/2002	AY676383	2002	ThD3_0328_02
		AY676408/Thailand/2000	AY676408	2000	ThD3_1017_00
		AY676406/Thailand/1998	AY676406	1998	ThD3_1283_98
		AY676386/Thailand/1992	AY676386	1992	ThD3_0240_92
		L11441/Thailand/1986	L11441	1986	D86-007
		AY676418/Thailand/1985	AY676418	1985	ThD3_0220_85
		AY676384/Thailand/1977	AY676384	1977	ThD3_285M_77
		L11620/Thailand/1973	L11620	1973	CH53489D73-1
	Vietnam	JF968110/Vietnam/2010	JF968110	2010	D3/Vietnam/1011aTw
		JN376776/Vietnam/2009	JN376776	2009	VL-M-1263/09
		JN376774/Vietnam/2007	JN376774	2007	AG-M-1639/07
		JN376773/Vietnam/2005	JN376773	2005	DT-M-2850/05
		JN376771/Vietnam/2003	JN376771	2003	BR-M-7144/03
		JN376769/Vietnam/2001	JN376769	2001	KG-M-2725/01
		DQ518654/Vietnam/1998	DQ518654	1998	Viet9809a/T
	Cambodia	KF543274/Cambodia/2011	KF543274	2011	V0907330
		JF968058/Cambodia/2008	JF968058	2008	D3/Cambodia/0808aTw
		EU367962/Cambodia/2007	EU367962	2007	07CHLS001
	Philippines	JN575570/Philippines/2010	JN575570	2010	Philippines 2010
		JF968061/Philippines/2008	JF968061	2008	D3/Philippines/0808cTw
		DQ518673/Philippines/2005	DQ518673	2005	Phil0508a/Tw
		DQ518669/Philippines/1998	DQ518669	1998	Phil9809a/Tw
		L11423/Philippines/1956	L11423	1956	H87
	Indonesia	KC589012/Indonesia/2012	KC589012	2012	SMG-SE005
		JN575561/Indonesia/2010	JN575561	2010	Bali 2010c
		AY858042/Indonesia/2004	AY858042	2004	KJ30i
		DQ675520/Indonesia/1998	DQ675520	1998	98TW182
	Malaysia	JF968112/Malaysia/2010	JF968112	2010	D3/Malaysia/1012bTw
		JF968068/Malaysia/2008	JF968068	2008	D3/Malaysia/0811aTw
		EU448439/Malaysia/2006	EU448439	2006	D3/Malaysia/0610aTw
		FM986662/Malaysia/1997	FM986662	1997	MY18-3
		AY338494/Malaysia/1994	AY338494	1994	LN7933
		JN030188/Singapore/2010	JN030188	2010	SG(EHI)D3/22866Y10
		JN030195/Singapore/2009	JN030195	2009	SG(EHI)D3/72901Y09
		EU081203/Singapore/2005	EU081203	2005	D3/SG/05K3324DK1/2005
		JN575572/Papua New Guinea/2010	JN575572	2010	PNG 2010a
	India	JQ686080/India/2010	JQ686080	2010	NIV_1029582
		JQ686072/India/2009	JQ686072	2009	NIV_0948350
		FJ644564/India/2007	FJ644564	2007	ND143
		AY770511/India/2003	AY770511	2003	GWL-25
		L11424/India/1984	L11424	1984	1416
	Bhutan	FJ606712/Bhutan/2007	FJ606712	2007	SV0786_07
	Yemen	HQ336219/Yemen/2010	HQ336219	2010	Yemen 2010
	Bangladesh	JF968085/Bangladesh/2009	JF968085	2009	D3/Bangladesh/0911aTw
		AY656671/Bangladesh/2000	AY656671	2000	165
		DQ518665/Bangladesh/2001	DQ518665	2001	Bang0108a/Tw
	Sri Lanka	L11438/Sri Lanka/1991	L11438	1991	Sri Lanka 1991
		KF955476/Sri Lanka/1983	KF955476	1983	DENV-3/LK/BID-V2415/1983
Oceania	Australia	JN575579/Australia/2009	JN575579	2009	Townsville 2009
		JN406514/Australia/1998	JN406514	1998	Cairns 98
	New Caledonia	JQ920485/New Caledonia/1996	JQ920485	1996	NC96/211096-4631
	French Polynesia	AY744685/French Polynesia/1994	AY744685	1994	PF94/136116
		AY744677/French Polynesia/1989	AY744677	1989	PF89/27643

(continued)

Supplemental Table 2

Continued

Continent	Geography	DENVs strains in this study	GenBank	Collection year	Strain
North America	United States	EU596492/USA/2007	EU596492	2007	DENV-3/US/BID-V1415/2007
		FJ390372/USA/2003	FJ390372	2003	DENV-3/US/BID-V1730/2003
		EU726768/USA/2000	EU726768	2000	DENV-3/US/BID-V1465/2000
		EU482558/USA/1998	EU482558	1998	DENV-3/US/BID-V1049/1998
		L11433/Puerto Rico/1963	L11433	1963	PR6
	Nicaragua	JF937623/Nicaragua/2010	JF937623	2010	DENV-3/NI/BID-V5514/2010
		FJ850052/Nicaragua/2008	FJ850052	2008	DENV-3/NI/BID-V2644/200
	Mexico	FJ898442/Mexico/2007	FJ898442	2007	DENV-3/MX/BID-V2989/2007
		FJ898440/Mexico/2003	FJ898440	2003	DENV-3/MX/BID-V2985/2003
	Costa Rica	JF804039/Costa Rica/1995	JF804039	1995	CR/DB026/1995
	EI Salvador	JX891665/EI Salvador/2012	JX891665	2012	SV/DB114/2012
South America	Venezuela	FJ639827/Venezuela/2008	FJ639827	2008	DENV-3/VE/BID-V2268/2008
		FJ639810/Venezuela/2005	FJ639810	2005	DENV-3/VE/BID-V2247/2005
		EU482614/Venezuela/2001	EU482614	2001	DENV-3/VE/BID-V913/2001
	Brazil	FJ850094/Brazil/2008	FJ850094	2008	DENV-3/BR/BID-V2403/2008
		GQ8685474/Brazil/2006	GQ868547	2006	DENV-3/BR/BID-V3444/2006
		FJ898446/Brazil/2001	FJ898446	2001	DENV-3/BR/BID-V2977/2001
	Peru	DQ177897/Peru/2005	DQ177897	2005	MFI624/Iquitos-Jan.2005
Africa	Tanzania	AB549332/Tanzania/2010	AB549332	2010	D3/Hu/Tanzania/08/2010NIID
	Mozambique	FJ882575/Mozambique/1985	FJ882575	1985	DENV-3/MZ/BID-V2418/1985
	Somalia	KC848585/Somalia/2011	KC848585	2011	SO/DB127/2011

DENV = dengue virus.
